# Tumor Primary Site and Histology Subtypes Role in Radiotherapeutic Management of Brain Metastases

**DOI:** 10.3389/fonc.2020.00781

**Published:** 2020-07-07

**Authors:** Muhammad Khan, Sumbal Arooj, Rong Li, Yunhong Tian, Jian Zhang, Jie Lin, Yingying Liang, Anan Xu, Ronghui Zheng, Mengzhong Liu, Yawei Yuan

**Affiliations:** ^1^Department of Radiation Oncology, State Key Laboratory of Respiratory Disease, Affiliated Cancer Hospital & Institute of Guangzhou Medical University, Guangzhou Medical University, Guangzhou, China; ^2^Department of Oncology, First affiliated Hospital of Anhui Medical University, Hefei, China; ^3^Department of Biochemistry, University of Sialkot, Sialkot, Pakistan; ^4^Department of Radiation Oncology, Sun Yat-sen University Cancer Center, Guangzhou, China

**Keywords:** whole brain radiotherapy (WBRT), stereotactic radiosurgery (SRS), brain metastases (BM), tumor control (TC), overall survival (OS), primary histology

## Abstract

Randomized controlled trials have failed to report any survival advantage for WBRT combined with SRS in the management of brain metastases, despite the enhanced local and distant control in comparison to each treatment alone. Literature review have revealed important role of primary histology of the tumor when dealing with brain metastases. NSCLC responds better to combined approach even when there was only single brain metastasis present while breast cancer has registered better survival with SRS alone probably due to better response of primary tumor to advancement in surgical and chemotherapeutic agents. Furthermore, mutation status (EGFR/ALK) in lung cancer and receptor status (ER/PR/HER2) in breast cancer also exhibit diversity in their response to radiotherapy. Radioresistant tumors like renal cell carcinoma and melanoma brain metastases have achieved better results when treated with SRS alone. Secondly, single brain metastasis may benefit from local and distant brain control achieved with combined treatment. These diverse outcomes suggest a primary histology-based analysis of the radiotherapy regimens (WBRT, SRS, or their combination) would more ideally establish the role of radiotherapy in the management of brain metastases. Molecularly targeted therapeutic and immunotherapeutic agents have revealed synergism with radiation therapy particularly SRS in treating cancer patients with brain metastases. Clinical updates in this regard have also been reviewed.

## Rationale

Brain metastasis is associated with worst prognosis and considered a major cause of cancer morbidity and mortality (1). Radiation therapy has long been the mainstay of treatment for brain metastases and is still contributing to this group of patients (2). A number of randomized controlled trials were conducted comparing different types of radiation therapies. Whole brain radiotherapy alone or in combination with stereotactic radiosurgery or stereotactic radiosurgery alone were the main components of investigation in these trials. These modes of treatments were compared for their safety and efficacy in terms of control and overall survival (Table 1) (3–8). Combined approach has revealed better brain control but no survival advantage (56). However, a survival advantage was demonstrated for combined approach when secondary analyses were restricted to patients with favorable prognosis (13, 14). Interesting point to note here is, these analyses were restricted to NSCLC primary histology (13, 14). Another aspect is if this benefit in control achieved with combined treatment could also lead to survival advantage in patients with solitary brain metastases. In fact, surge in survival with combined approach was reported when single brain metastases were considered only (4). These observations may make one think that the better local and distant control associated with combined approach might lead to survival advantage if a more dynamic selection of patient is exercised. Here, we report some of the related evidence highlighting these two points: a possible miscalculation in designing randomized controlled trial for this group of patients. The scope of this paper is restricted to the three radiotherapeutic treatment regimens (WBRT alone, SRS alone and WBRT plus SRS) in the treatment of brain metastases. In addition, relevant advancements in the field of targeted therapy and immunotherapy alone or in combination with radiotherapy have also been reviewed.

**Table 1:** General characteristics and main outcomes of the studies.

	**Studies**	**Study type and no. of BM**	**No. of patients**	**Treatments and comparison**	**Primary histology**	**Tumor control (months)**	**Survival (OS) (months)**	**Prognostic factors (OS)**	
								**Univariate**	**Multivariate**
Primary randomized controlled trials	Kondziolka et al. (3)	RCT 2–4 BMs	27 13/14	WBRT vs. WBRT+SRS	Lung, Melanoma, RCC, Breast, Other	LC: 36 vs. 6 (*p* = 0.0005)	MST: 7.5 vs. 11 (*p* = 0.22) breast 11 vs. lung 11 vs. melanoma 5.5 (*p* = 0.17)	Extent of extracranial disease	–
	Andrews et al. (4)	RCT 1–3 BMs	331 167/164	WBRT vs. WBRT+SRS	Breast, Lung (Squamous, Adenocarcinoma, Large cell, Small cell), Melanoma, Renal, Other	LC: *p =* 0·0132 (Favors WBRT + SRS)	MST: 5.7 vs. 6.5 (*p =* 0·1356) Single BM (6·5 vs. 4·9, *p =* 0·0393) Squamous NSCLC (5·9 vs. 3·9, *p =* 0·0508) **(Favors WBRT + SRS)**	Single metastases, RPA class 1, largest metastasis was > 2 cm in diameter	RPA 1 and type of tumor (Lung primary)
	Aoyama et al. (5)	RCT 1–4 BMs	132 67/65	WBRT+SRS vs. SRS	Breast, Lung, Colorectal Renal, Other	12 month BTRR: 46.8% 76.4% (*p* < 0.001)	MST: 8.0 (0.5-57.0) vs. 7.5 (0.8–58.7) (*p* = 0.42)	Primary tumor status (stable), Extracranial metastases (stable), RPA 1, KPS (90–100)	Age (<65 y), Primary tumor status (stable), Extracranial metastases (stable), KPS score (90–100)
	El Gantery et al. (6)	RCT 1–3 BMs	60 21/18/21	WBRT+SRS vs. SRS vs. WBRT	–	LC: 10 vs. 6 vs. 5 (*p* = 0.04)	NS - Largest brain metastases = 3 cm in diameter: 15 vs. 8 vs. 5, *p =* 0.002 - Controlled primary: 12 vs. 8 vs. 5.5, *p =* 0.027 **(Favors WBRT + SRS)**	Single brain metastasis	–
	Brown et al. (7)	RCT 1–3 BMs	213 111/102	SRS vs. WBRT+SRS	Breast, Colorectal, Lung, Skin/melanoma, Bladder, Kidney, Gynecologic, Other	ICF: HR: 3.6; 95% CI, 2.2-5.9; (*p* < 0.001)	OS: HR: 1.02; 95% CI, 0.75–1.38; (*p =* 0.92)	–	–
	Chougule et al. (8)	RCT multiple	109 36/37/31	SRS vs. WBRT vs. WBRT+SRS	Breast, Lung, colorectal	LC: 87 vs. 91 vs. 62%	MST: 7 vs. 5 vs. 9 *p =* NS - Breast 9.5 vs. Colorectal 7 vs. Lung 6	–	–
Retrospective studies	Sanghavi et al. (9)	Retrospective	1,702 502/1200	WBRT+SRS vs. WBRT	Lung, breast, melanoma and others	–	RPA I = 16.1 vs. 7.1 RPA II = 10.3 vs. 4.2 RPA III = 8.7 vs. 2.3 (*p* < 0.05)	KPS, a controlled primary, absence of extracranial metastases, and RPA class	–
	Sneed et al. (10)	Retrospective	569 268/301	SRS vs. WBRT+SRS	Breast, Kidney, lung, melanoma and others	–	HR: 1.07 (0.89–1.27), *p =* 0.49	KPS, Extracranial metastases, Control of the primary, Number of metastases	–
	Frazier et al. (11)	Retrospective	237 192/45	SRS vs. WBRT+SRS	Breast, Melanoma, NSCLC, Renal, Other	5.9 (4.6–7.3) vs. 6.7 (4.0–12.1) (*p* = 0.22)	14.6 (11.4–19.1) vs. 10.8 (6.2–18.0) (*p* = 0.31)	KPS >70, **histology of breast cancer**, smaller tumor volume, and age <65 years	–
	Elaimy et al. (12)	Retrospective	275 65/15/48/ 19/117/11	SRS/S+SRS/ WBRT+SRS/S +WBRT+SRS /WBRT/ S+WBRT	NSCLC, SCLC, Breast, Melanoma, RCC, Other	-	SRS vs. WBRT (HR:1.94; 95% CI: 1.37–2.73, *p* < 0.001) SRS vs. WBRT+SRS (HR:0.99; 95% CI: 0.93–1.05, *p =* 0.660)	ECOG-PS, Primary histology - NSCLC vs. Melanoma & RCC (HR:1.17; 95% CI: 1.06-1.3, *p* < 0.001) - NSCLC vs. Breast (HR:0.87; 95% CI: 0.78-0.96, *p* < 0.001)	–
Lung cancer	Sperduto et al. (13)	Secondary analysis (RCT)	252 126/126	WBRT+SRS vs. WBRT	Lung, gastrointestinal, renal cancers and melanoma	–	HR: 1.0; 95% CI: 0.8–1.4, *p* = 0.78)	MST: 21.0 vs. 10.3, (*P* = 0.05) **(GPA 3.5–4.0)**	–
	Aoyama et al. (14)	Secondary analysis (RCT)	88 45/43	SRS vs. WBRT+SRS	NSCLC	HR: 5.01 (2.44–11.11, *p* < 0.001)	HR: 1.33 (0.85–2.08, *p =* 0.20)	HR: 1.92; 95% CI, 1.01–3.78, *p* = 0.04) **DS GPA 2.5–4.0 group**	–
	Churilla et al. (15)	Secondary analysis (RCT)	127 70/57	SRS vs. SRS+WBRT	NSCLC	HR: 4.11 (2.11–8.00), *p* < 0.001	HR: 0.98 (0.66–1.46), *p =* 0.92		–
	Li et al. (16)	Retrospective Single BMs	70 29/23/18	WBRT/SRS vs. SRS+WBRT	Lung (SCLC, NSCLC)	- FFLP: 3.97 ± 0.33 vs. 6.85 ± 0.50 vs. 8.56 ± 1.36 (*P* < 0.0001) - FFNBM: 4.07 ± 0.32 vs. 6.74 ± 0.52 vs. 8.56 ± 1.36 (*P* < 0.0001) - SRS vs. SRS+WBRT (*p =* 0.0392)	MST: 5.67 ± 0.38, 9.33 ± 0.59, and 10.64 ± 1.54, (*P* < 0.0001) SRS vs. SRS+WBRT (*p =* 0.7079)	Tumor volume, the absence of active extra-cranial disease, treatment methods, and worst pattern of enhancement	–
	Sperduto et al. (17)	Prospective multiple	1,888 815/396/342	WBRT vs. SRS/WBRT +SRS	NSCLC	–	HR: 0.62;0.51–0.75, *p* < 0.0001 HR: 0.53;0.45–0.63, *p* < 0.0001	–	Age, KPS, ECM, No. of BMs
	Lin et al. (18)	Retrospective multiple	20,396 20241/155	WBRT vs. WBRT+SRS	NSCLC	–	HR: 0.49 (0.36–0.66), *p* < 0.0001	–	–
	Minniti et al. (19)	Prospective 2–3 BMs	122 66/66	WBRT vs. WBRT+SRS	NSCLC	- LC 6 month: 90 vs. 100% - 12 month: 47 vs. 93% - BC 6 month: 75 vs. 82% 12 month: 18 vs. 42% (*p =* 0.001)	MST: 7.2 vs. 10.3, *p =* 0.005	Stable extracranial disease and KPS	–
	Marko et al. (20)	Prospective multiple	162 26/121/15	SRS vs. WBRT vs. WBRT+SRS	NSCLC	–	MST: 12.32 vs. 12.25 vs. 12.74, (*p =* 0.98, 0.62, 0.91)	–	–
	Abacioglu et al. (21)	Prospective multiple	100 (22/78)	SRS vs. WBRT+SRS	NSCLC (Adenocarcinoma Squamous cell carcinoma Unclassified NSCLC)	–	MST: 8 vs. 9, *p =* 0.757	**Adenocarcinoma histology**, KPS score ≥ 80, 1–3 metastases and tumor diameter < 2 cm	–
	Sun et al. (22)	Prospective multiple	82 (33/49)	WBRT+SRS vs. WBRT	SCLC	–	MST: 13.4 vs. 8.5 months; *p* = 0.004 OS rate at 6 m: 84.5 vs. 59.8% 12 m: 62.7 vs. 29.9% 24 m: 21.5 vs. 9.6% (*p =* 0.004)	Limited number (1 to 3) of BMs, KPS ≥ 70, asymptomatic BMs, controlled extracranial diseases, and maximum diameter of the largest tumor ≤ 2.0 cm	–
	Mansour and Shawky (23)	Prospective multiple	36	SRS+WBRT	SCLC	–	MST: 13.5 OS rate at 6m: 84.5% 12m: 62.7% 24m: 21.5%	KPS, single BMs, controlled extracranial diseases, ≤ 2 cm maximum diameter of the largest BMs tumor and asymptomatic BMs	≤ 2 cm maximum diameter of the largest BMs tumor
	Wegner et al. (24)	Prospective multiple	44 (6/38)	WBRT+SRS vs. SRS (prior WBRT or PCI = 30)	SCLC	ALC at 6 m: 90%, 12 m: 86%	MST: 14 vs. 6 (*p* = 0.04)	–	–
	Sperduto et al. (17)	Prospective multiple	268 (247/21)	WBRT vs. WBRT+SRS	SCLC	–	MST: 3.87 vs. 15.23, *p =* 0.003	–	KPS, age, ECM, No. of BMs
Breast cancer	Caballero et al. (25)	Retrospective	310	SRS after prior WBRT	90 breast, 113 NSCLC, 31 SCLC, 42 melanoma, and 34 miscellaneous	–	MST: 8.4 (11.4 vs. 8.1 vs. 7.2)	Breast; age <50 years, smaller total target volume, and longer interval from WBRT to SRS NSCLC; controlled primary tumor, and number of BM, melanoma; smaller total target volume	Breast; age <50 years, smaller total target volume, and longer interval from WBRT to SRS NSCLC; number of BM, KPS, and controlled primary Melanoma; smaller total target volume
	Firlik et al. (26)	Retrospective		SRS vs. WBRT+SRS	Breast cancer	93%	*P =* 0.20	Tumor volume and Solitary metastasis	–
	Muacevic et al. (27)	Retrospective		SRS vs. WBRT+SRS	Breast cancer		9.5 ± 1.4 vs. 11.4 ± 3.5, *p =* 0.7	KPS and RPA	–
	Kased et al. (28)	Retrospective		SRS vs. WBRT+SRS	Breast cancer	MBFFP: 8.6 vs. 10.5, *p* = 0.75	MST: 17.1 vs. 15.9, *p =* 0.20	Age <50 y KPS > 70 Primary controlled, ER positive Overexpression, Her2/neu overexpression.	–
	Sperduto et al. (17)	Retrospective multiple	642 277/141/ 123	WBRT vs. SRS/WBRT +SRS	Breast cancer	–	HR: 0.75;0.54–1.04, *p =* 0.088 HR: 0.72;0.53–0.98, *p =* 0.035	NA	KPS
	Jaboin et al. (29)	Retrospective	100 26/25/37	SRS vs. SRS+WBRT vs. WBRT+ (salvage)SRS	Breast (luminal A, luminal B, HER2/neu, basal, unknown)	–	MST: 12.4 vs. 12.2 vs. 9.5, *p =* NS	Age, stage and number of lesions, CNS failure	–
	Perez et al. (30)	Retrospective	231 66/165	SRS vs. SRS+WBRT	Breast cancer	–	HR: 1.78;1.06–2.99, *p =* 0.03 (multivariate)	Controlled systemic disease, adjuvant chemotherapy, and RPA	–
	Sperduto et al. (31)	Retrospective	383	SRS/WBRT/ WBRT+SRS/ S+WBRT/S+ SRS+WBRT/ S+SRS	Basal (TN) Luminal A (ER/PR(+)/HER2(–) HER2(+)/ER/PR(–) Luminal B (TP)	–	MST: 7.3 (4.9–9.5) 10.0 (7.4–19.5) 17.9 (13.4–22.9) 22.9 (16.1–29.5) *p* < 0.01	–	–
	Cho et al. (32)	Retrospective	131 79/43+4/5	SRS vs. WBRT+ salvage SRS vs. WBRT+SRS boost vs. S+SRS boost	ER(+)/HER2(–); 41(31%), ER(+)/HER2(+); 30 (23%), ER(–)/HER2(+); 23 (18%), and ER(–)/HER2(–); 28 (21%) (TNBC).	TNBC vs. ER(+)/HER2(–); HR:3.12 (*p* < 0.001) (retreatment or death)	SRS vs. WBRT+SRS: *HR* = 1.18, *p* = 0.4 MST: 16 vs. 26 vs. 23, vs. 7 (*p* < 0.001)	–	–
	Xu et al. (33)	Retrospective	264 HER2known /unknown (172/92)	SRS vs. S/WBRT 162/49+214	Breast cancer HER2+ vs. HER2–172 (82/90)	–	SRS vs. S/WBRT: 96.6 vs. 106.5, *p =* 0.73 MST: OS:105.7 vs. 74.3, *p* < 0.01 Survival after SRS: 31.3 vs. 14.1, *p* < 0.01	HER2+; HR:0.66, *p =* 0.021 Age >45 y, Estrogen receptor positive, Progesterone receptor positive	HER2+; HR:0.18, *p* < 0.001
	Xu, et al. (34)	Retrospective	103 (SRS = 27, WBRT+SRS = 59 S+SRS = 9, S+WBRT+ SRS = 8)	SRS vs. WBRT+SRS	Breast cancer (Triple negative, Non–triple negative, ER+, PR+, HER2+) (T*N =* 24/Non–T *N =* 79	- OLC: 90/3% - ATC rate at 6 m: 96.5% - at 12 m: 92.2% - at 24 m:83.3%	SRS vs. WBRT+SRS; *p =* 0.797 Non-TN vs. TN; MST: 43 (27.3–58.7) vs. 82 (66.3–97.7), *p =* 0.042	Non-TN vs. TN; HR:0.461 (0.279–0.763), *p =* 0.003 HER2+ vs. HER2–; HR:0.629 (0.405–0.975), *p =* 0.038	Non-TN status and lower recursive partitioning analysis class
Radioresistant histology	Lwu et al. (35)	Retrospective	103 34/56	SRS alone vs. SRS + prior WBRT	41 RCC, 62 Melanoma	ALC at 6m: 89% 12 m: 84% 18: 76% 24 m:61% - LC at 12 m: 91% (RCC) and 75% (melanoma)	HR: 0.98 (0.30–3.26), *p =* 0.98 - Melanoma vs. RCC; HR: 3.48 (1.08–11.23), *p =* 0.04)	Tumor volume, Primary tumor	–
	Brown et al. (36)	Retrospective	41	SRS vs. SRS+WBRT boost	16 RCC, 23 melanoma, 2 sarcoma	LF; 12% DBF; 54% **-SRS+WBRT vs. SRS:** - ALC at 6 month; 100 vs. 85%, *p =* 0.018 - DBF rate at 6-m; 17 vs. 64%, *p =* 0.0027	MST: 14.2 - RCC vs. melanoma; 17.8 vs. 9.7, *p =* 0.12	Systemic disease status, RPA	RPA, histological diagnosis of primary tumor
	Manon et al. (37)	Retrospective	31	SRS	Melanoma 14, Sarcoma 3, RCC 14	ICF at 3m; 25.8% - at 6 m; 48.3%	8.3 months (95% CI, 7.4 to 12.2).	–	–
	Chang et al. (38)	Retrospective	189	SRS	103 melanoma, 77 RCC, 9 sarcoma	1-year AFFP: 64% RCC; 47% melanoma; 0% sarcoma (*P* < 0.001)	MST: 7.5 1-year SR: 40% RCC; 25% melanoma; 22% sarcoma (*P* = 0.0354)	–	–
Renal cell carcinoma	Wronski, M., et al. (39)	Retrospective	119	WBRT	RCC		MST: 4.4	–	single brain metastasis, lack of distant metastases at the time of diagnosis, and tumor diameter < or = 2 cm
	Takashi et al. (40)	Retrospective	69	SRS	RCC	82.6%	MST: 9.5	–	Number of lesions, KPS, RPA, and the interval from diagnosis of RCC to brain metastasis
	Jasonet al. (41)	Retrospective	69	SRS	RCC	ALC; 94%	MST: 6	–	Age, preoperative KPS score, radiosurgical dose to the tumor margin, maximal radiosurgical dose, treatment iso-dose, time from diagnosis of renal cell cancer to the development of brain metastasis
	Goyal et al. (42)	Retrospective	29 (13/16)	SRS vs. SRS+WBRT	RCC	-DBF: 33 vs. 25% - LC: 2/18 vs. 2/29, *p =* NS	MST: 5.2 vs. 6.8, NS	–	–
	Mori et al. (43)	Retrospective multiple	25 12/13	SRS vs. SRS+WBRT	RCC		MST: 11 SRS vs. SRS+WBRT; *p =* 0.35	Age, good KPS at the time of radiosurgery, nephrectomy prior to radiosurgery	Age, lack of active systemic disease, use of chemotherapy and/or immunotherapy after SR
	Ippen et al. (44)	Retrospective multiple	66 36/24/6	SRS vs. S+SRS vs. SRS+WBRT	RCC	−1-year LC: 84%, 94%, and 88%, *p* = 0.445 - DTC: Prior WBRT vs. others (*p =* 0.007)	OS: 13.9, 21.9, 5.9 - Prior WBRT was associated with worst OS (uni/multivariate)	Age, prior surgery, RPA, KPS, SIR, BSBM, number of brain metastases, initial tumor volume, and Ds-GPA	Age, RPA, KPS, and the initial number of brain metastases, prior surgery
	Fokas et al. (45)	Retrospective 1–3 (SRS/SRS+ WBRT) Multiple (WBRT)	88 51/17/20	SRS vs. SRS+WBRT vs. WBRT	RCC	1-, 2-, 3-year IC rates; 42%, 29%, 22%	MST: 12 vs. 16 vs. 2 -SRS/SRS+WBRT vs. WBRT; *p* < 0.001	Age, lack of extracranial metastases, RPA, SRS, SRS + WBRT	lack of extracerebral metastases, RPA, SRS, SRS + WBRT
	Bates et al. (46)	Retrospective multiple	25 9/11/5	SRS vs. WBRT vs. SRS+WBRT	RCC	BPFS; 8.3 vs. 2.5 (*p =* 0.38) vs. 4.5 (*p =* 0.65).	OS; 8.3 vs. 2.8 (*p =* 0.82) vs.8.5 (*p =* 0.65)	Age, sex, KPS, presence of extracranial metastases, history of smoking, alcohol consumption, DS-GPA, use of surgery, multiple intracranial metastases	DS-GPA score
Melanoma	Hauswald et al. (47)	Retrospective	87	WBRT	Melanoma	–	MST: 3.5 OSR: - at 6 m: 29.2% - at 12 m: 16.5%	DS-GPA, RPA	Total treatment dose, surgical resection, GPA
	Noël et al. (48)	Retrospective	25	SRS	Melanoma	3-, 6- and 12-m LC rates; 95 ± 3, 90 ± 5 and 84 ± 7%	MST: 8 months 3-, 6- and 12-m OS rates; 75 ± 9, 53 ± 10, and 29 ± 10%	Extracranial controlled disease, SIR	-
	Seung et al. (49)	Retrospective	55 11/28/16	WBRT+SRS, SRS, WBRT+SRS (salvage)	Melanoma	6 month and 1-year actuarial freedom from progression rates of 89% and 77%	35 wks	Total target volume treated	–
	Mathieu et al. (50)	Retrospective	244 115/110/53	SRS (prior WBRT /prior surgery)	Melanoma	LC: 30.9% DC: 41.7%	MST: 5.3	Age, Extracranial disease status, RPA, KPS, Number of metastases, Single or multiple metastasis, WBRT at any time	Active extracranial disease, KPS, multiple metastases, tumor volume >8 cm^3^, cerebellar metastases
	Yu et al. (51)	Retrospective	122	SRS vs. SRS+WBRT 39 (32%) WBRT	Melanoma	–	MST: 7.0	total intracranial tumor volume <3 cm^3^, inactive systemic disease	–
	Selek, U., et al. (52)	Retrospectiv3	103 61/12/30	RS, SRS+WBRT, WBRT+ SRS (salvage)	Melanoma	−1-year LC; 49% - 1-year DF; 14.7%.	1-year OS: 25.2%	Score Index for Radiosurgery (SIR)	
	Dyer et al. (53)	Retrospective	147	SRS/SRS+ WBRT/salvage WBRT	Melanoma	DICF: omission of up-front WBRT; HR: 2.24, *p* = 0.005	MST: 7.3 Omission of up-front WBRT; HR: 2.56, *p* = 0.08 (multivariate)	Extensive extracranial metastases, KPS, multiple brain metastases	Extensive extracranial metastases, KPS
	Bagshaw et al. (54)	Retrospective	185 154/51/31	SRS/salvage WBRT /SRS+WBRT	Melanoma	MTTLF: 23.4	MST: 7.8	–	–
	Radbillet al. (55)	Retrospective	51 32/8/8/2	SRS/SRS+ WBRT/SRS+S /SRS+WBRT +S	Melanoma	ALC - at 26 wks: 66% -at 52 wks: 56% ADC - at 26 wks: 46% - at 52 wks: 25%	OS rate - at 12 wks; 71% - at 26 wks;51%, - at 52 wks; 30%	–	RPA I, Treatment of infratentorial lesion, Multiple lesions present (categoric) Initial WBRT with radiosurgery; HR:1.08 (0.39–2.98), *p =* 0.88

*OS, overall survival; MST, median survival time; LC, local control; DF, distant failure; DC, distant control; ICF, intrcranial failure; MTTLF, median time to local failure; MTTDF, median time to distant failure; ALC, actuarial local control; DICF, distant intracranial failure; BPFS, brain progression free survival; IC, intracranial; DTC, distant tumor control; DBF, distant brain failure; AFFP, actuarial freedom from progression; FFNBM, free from new brain meatsases; RPA, recursive partitioning analysis; DS-GPA, Diagnosis-specific graded prognostic assessment; WBRT, whole brain radiotherapy; SRS, stereotactic radiosurgery; S, surgery; RCT, randomized controlled trial; NSCLC, non-small cell lung cancer, SCLC, small cell lung carcinoma; wks, weeks; m, months; NS, not significant; ER+, estrogen receptor positive; PR+, progesterone receptor positive; HER2+, human epidermal growth factor receptor 2; TN, triple negative; TNBC, triple negative breast cancer*.

## Primary Cancer Histology

**Figure 1:**
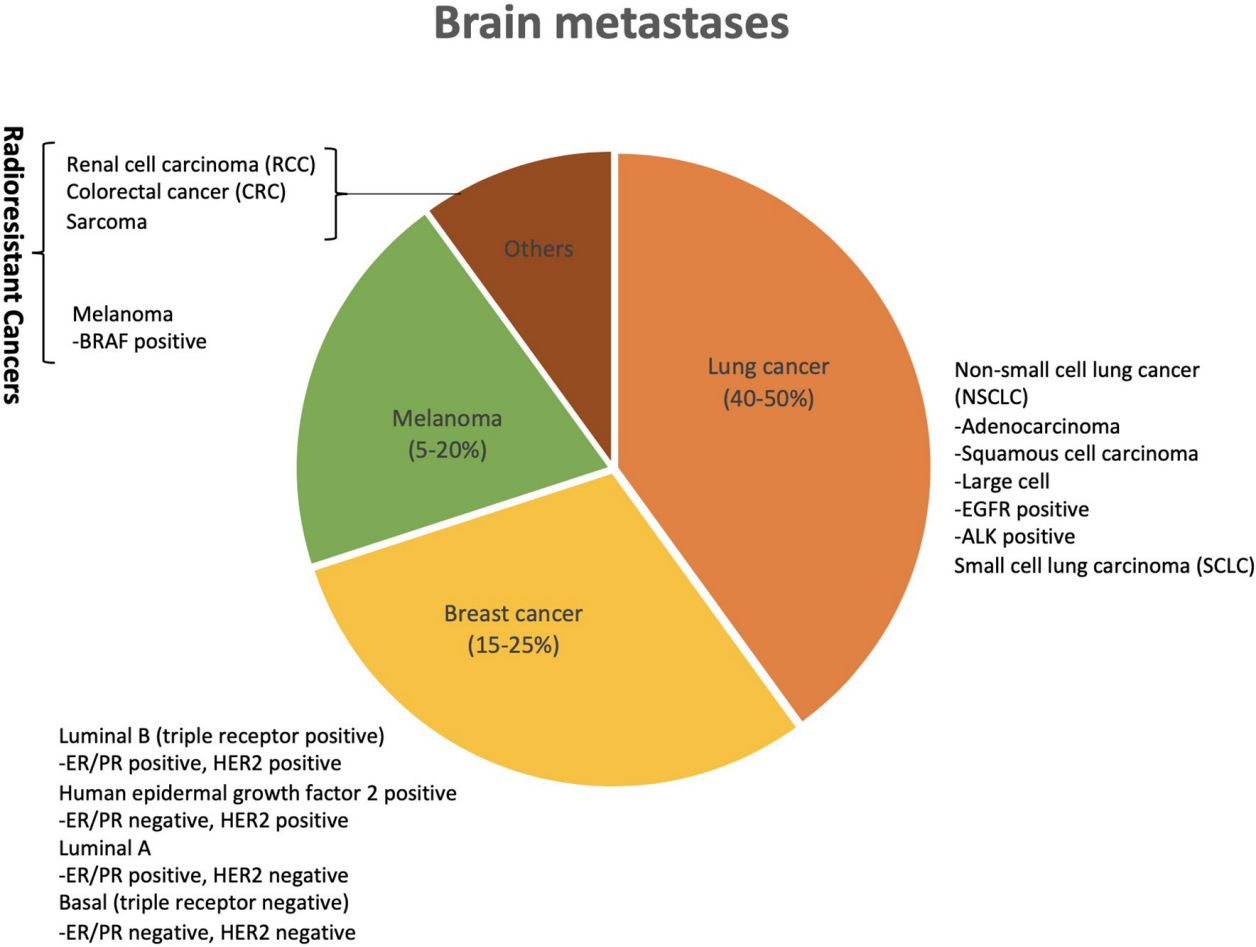
Primary cancer sites with corresponding frequencies of causing brain metastases. Lung cancer is the most frequent to cause brain metastases followed by breast, melanoma and renal cell carcinoma. Histology subtypes and mutation status (EGFR/ALK) in lung cancer, and receptor status (+/–) in breast cancer have also shown relevance when it comes to their response in terms of brain control and survival to radiation therapy in the form of WBRT (whole brain radiotherapy), SRS (stereotactic radiosurgery) and combination of both (WBRT plus SRS).

Brain metastases originate from various primary cancers with different frequency and propensity. Lung cancer (40–50%) being the most frequent followed by breast (15–25%), or melanoma (5–20%), and to a smaller extent from renal cell carcinoma, colorectal cancer and sarcoma (Figure 1) (57). It has been suggested that primary cancer histology may play an important role in determining the survival due to its response to different treatments (radiation or chemotherapeutic), propensity to metastasize and aggressiveness (11).

### Lung Cancer

Primary site and histology subtypes have been regarded essential in deriving survival advantage from radiation therapy in these patients (11, 12). Lung cancer histology is broadly divided into two main types; non-small cell lung cancer (NSCLC) and small cell lung cancer (SCLC). NSCLC constitute 80–85% of overall lung cancer cases. Further subclasses of NSCLC include adenocarcinoma (50%), squamous cell carcinomas (30%) and large cell carcinomas (58, 59). So far, the trials as well as several other retrospective studies had pooled together patients with brain metastases originating from different primary sites and histology including the ones resistant to radiation therapy such as malignant renal cell carcinoma and melanoma (3–12). Lung cancer has been the most frequent histology in these trials (3–8). Andrew et al.'s RCT of 331 brain metastatic patients revealed superior survival in patients with squamous NSCLC for the treatment difference favoring WBRT plus SRS (*p* = 0.0508). This result was not reciprocated for adenocarcinoma subtype (4). Secondary analysis of this trial which included 252 patients evaluated by GPA (graded prognostic assessment) also revealed survival benefit in patients (primarily lung cancer 211/252) with favorable prognosis (DS-GPA 3.5–4.0) assessed by diagnosis specific graded post-stratification assessment (median survival time [MST] for WBRT + SRS vs. WBRT alone was 21.0 vs. 10.3 months, *P* = 0.05) (13). Secondary analysis of the JROSG 99-1 Randomized Clinical Trial, which compare WBRT plus SRS to SRS alone in patients with NSCLC and prognostic score of DS-GPA 2.5–4.0 (*n* = 47) demonstrated better survival with combined treatment (median survival time [MST] for WBRT + SRS vs. SRS alone was 16.7 vs. 10.6 months, *P* = 0.04) (14). Brain tumor recurrence rates were different between the two groups, suggesting additive WBRT had a more significant impact in the DS-GPA 2.5–4.0 group (HR, 8.31; 95% CI, 3.05–29.13) (*P* < 0.001) vs. the DS-GPA 0.5–2.0 group (HR, 3.57; 95% CI, 1.02–16.49) (*P* = 0.04). A similar secondary analysis of another trial, the NCCTG N0574 (Alliance) Randomized Controlled Trial, however, didn't reveal such improvement in a similar categorized group (DS-GPA 2.5–4.0) of recipients (*n* = 29) (15). It is noteworthy that of the 29 patients in this prognostic group of DS-GPA 2.5–4.0, 26 had achieved a score of DS-GPA 2.5–3.0, comparatively fewer than Aoyama et al. secondary analysis which included 37 patients (14, 15). The DS-GPA group (3.5–4.0) only had 3 patients and that too in SRS group only whereas Aoyama et al. contained 10 patients (SRS; 3 vs. WBRT+SRS; 7) (14, 15). Overall low number of participants and also the lack of patients achieving higher prognostic score in WBRT + SRS group make the Churilla et al. analysis less effectual (15). It's unfortunate that not much is available for analysis in this regard from the primary RCTs (3–8).

Apart from these primary trials, there are a number of retrospective studies in which combined treatment have resulted in better survival for patients with lung cancer brain metastases in comparison to WBRT alone (16–19, 22, 24). Li et al. showed significant improvement in survival for combined treatment in comparison to WBRT alone but not SRS alone for patients with single NSCLC brain metastasis (16). In a large retrospective study of patients with newly diagnosed brain metastases (*n* = 4,259) from various primary tumors reported significant survival benefit for SRS alone (HR: 0.62; 0.51–0.75, *p* < 0.0001) and WBRT plus SRS (HR: 0.53; 0.45–0.63, *p* < 0.0001) against WBRT alone for patients with brain metastases from NSCLC (*n* = 1,888) (17). Lin et al. concluded that GK radiosurgery combined with WBRT increased the survival of NSCLC patients with brain metastases (18). Minniti et al. also reported WBRT plus SRS to be a safe, minimally invasive and well-tolerated treatment for patients with up to three brain metastases from NSCLC resulting in longer survival and better disease control in comparison with WBRT alone (19). Marko et al. revealed numerically better survival (Kaplan–Meier survival means for the SRS only, WBRT only, WBRT plus SRS groups were 14.3, 14.8, and 19.1 months, respectively), however, statistically not significant (*P* = 0.143–0.159) (20). In multivariate analysis of a study of 100 patients and 184 brain metastases from NSCLC evaluating GKRS and prognostic factors for overall survival, adenocarcinoma histology revealed to be prognostic for survival. Addition of WBRT had no impact on survival in this study (21). Overall, SRS boost to WBRT reveals general superiority over WBRT alone but not SRS alone. In fact, omission of WBRT had no impact on survival (HR: 1.06; 0.90–1.26, *p* = 0.8084) for NSCLC patients with brain metastases in a comparative trial of WBRT plus optimum standard care (dexamethasone) compared to optimum standard care alone (60).

Small cell lung carcinoma constitutes a rather small group of lung cancer patients (15–20%) (58). Andrews et al. demonstrated survival benefit for patients with WBRT plus SRS in comparison to WBRT alone when analyses were restricted to 24 SCLC patients (*n* = 24) with 1 to 3 brain metastases (*p* = 0.039) (4). Sun et al. also revealed that WBRT plus a radiation boost (Cyberknife) was significantly associated with improved OS in patients with 1–3 SCLC brain metastases when compared to WBRT-alone (13.4 vs. 9.6 months, *p* = 0.022) (22). The 6-, 12-, and 24 month survival rates were also comparatively higher for combined treatment (84.5%, 62.7%, and 21.5 vs. 59.8%, 29.9%, 9.6%, *p* = 0.004). Similar survival rates (84.5, 62.7, and 21.5%) were repeated in study of 36 patients with SCLC brain metastases who had received WBRT boost (23). In comparison to SRS alone, WBRT with SRS boost had also shown survival efficacy as revealed in a study of 44 SCLC patients (WBRT + SRS vs. SRS; 14 vs. 6 m, *p* = 0.04). However, number of patients in combined group were comparatively smaller (*n* = 6) while patients in the SRS alone group had also received prior WBRT, PCI (prophylactic irradiation), or both (24). Sperduto et al. (17) retrospective analysis also contained a total of 299 patients with brain metastases from SCLC. A significant surge in survival (HR: 0.29; 0.13–0.66, *p* = 0.003) was derived with SRS boost to WBRT (*n* = 21) in contrast to WBRT alone (*n* = 247) (17). Elaimy et al. retrospective study disclosed patients with different primary histology responded differently to treatment. In univariate analysis, NSCLC was favored statistically in terms of survival than SCLC, and classified in the other primary histology group. Moreover, NSCLC patients derived significant survival benefit when compared to the combined melanoma and renal-cell carcinoma group on multivariate analysis (12). Overall, Lung cancer brain metastases seems to respond better to combined treatment and maybe recommended in patients with better prognostic standings.

There is considerable amount of progress in targeted therapy aimed at the mutation carrying by NSCLC patients such as EGFR (epidermal growth factor receptor) and ALK. Gefitinib and erlotinib (first generation EGFR targeting agents) have shown greater efficacy in the brain (61, 62). Studies that included randomized controlled trials, prospective trials and retrospective studies have reported conflicting results in terms of OS outcome for addition of radiation therapy to EGFR-TKIs in these patients (18, 61–77). However, Meta-analyses comprising most of these studies have suggested a significantly better survival associated with receiving additional radiation therapy (78–80). WBRT addition to EGFR-TKIs (gefitinib, erlotinib, and icotinib) have significantly increased survival for brain metastatic NSCLC patients with unknown EGFR status or without molecular selection (61, 64, 65, 71–73). Evidence included a randomized controlled trial revealing a median survival of 13.3 vs. 12.7 months (*P* < 0.05) (64). This advantage in OS has also been demonstrated in EGFR-mutated NSCLC patients (74–77). SRS plus EGFR-TKIs were reported to be equally effective in treating EGFR-mutated NSCLC patients against EGFR-TKIs alone in a retrospective study (70). However, Magnuson et al. study identified significant improvement for such patients either receiving upfront WBRT or SRS as compared to upfront EGFR-TKIs alone (76). Despite the fact that low prognostic patients were allocated to WBRT group, upfront WBRT followed by EGFR-TKIs produced better survival outcome (HR: 0.70; 0.50–0.98, *p* = 0.039) in comparison to upfront EGFR-TKIs. In the same study, SRS followed by EGFR-TKIs also derived better survival benefit as opposed to upfront EGFR-TKIs (HR: 0.39; 0.26–0.58, *p* = 0.001) (76). An RCT by Sperduto et al. revealed WBRT plus SRS in combination showed slight superior median survival, however not significant, for molecularly unselected NSCLC patients as opposed to addition of EGFR-TKIs to WBRT and SRS (13.4 vs. 6.1, *p* = NS) (81). Nonetheless, a retrospective study demonstrated efficacy of adding gefitinib to WBRT or WBRT plus SRS (18). In fact, efficacy was greater when WBRT plus SRS were applied. Addition of WBRT or WBRT + SRS to gefitinib reported significant improvement in survival for brain metastatic NSCLC patients opposite to WBRT alone (*p* < 0.0001). Furthermore, results of WBRT + SRS + gefitinib were superior to WBRT + SRS as well as WBRT + gefitinib (*p* < 0.001) (18). ALK inhibitors, particularly the second-generation agents alectinib and brigatinib, have also shown promising responses in the treatment of brain metastases (82). However, there is no study done for comparison of the combination with radiation therapy to ALK inhibitors alone. A prior RT delivery was shown to have a positive impact on brain efficacy of these agents. Immunotherapy agents like nivolumab and pembrolizumab are also being used rigorously making the treatment paradigm diverse (83). Furthermore, combination of these agents with WBRT plus SRS or WBRT alone or SRS alone would clearly establish the role of each radiation therapy for this group of patients.

### Breast Cancer

Like NSCLC histology, breast cancer as primary site for brain metastases has also demonstrated a distinct behavior in response to radiation therapy. A control rate between 90 and 94%, and median survival between 10 and 16 months, prognosis for breast cancer brain metastases appears to be superior in comparison to other histologic groups (84). Multivariate analysis of Elaimy et al. retrospective study showed breast cancer group was statistically better in term of survival to patients with NSCLC brain metastases (12). Frazier et al. identified primary breast cancer site to be prognostic of survival, which also included several other primary histologic sites including NSCLC, melanoma and renal cell carcinoma (11). Sperduto et al. retrospective analysis revealed superior survival for WBRT plus SRS vs. WBRT alone (HR: 0.72; 0.53–0.98, *p* = 0.035) in breast cancer patients (*n* = 642) (17). Radiosurgery as salvage therapy for tumor recurrence after fractionated WBRT has been helpful in prolonging the median survival from 3–5 to 10.3–14 months suggesting a better result for combined treatment compare to WBRT alone (84). Moreover, a longer interval from WBRT to SRS was identified on multivariate analyses as prognostic for breast cancer patients in a study of salvage SRS for BM after prior WBRT (25). Studies have shown breast cancer patients responding well to SRS alone and contribution of adding WBRT in this group of patients has not been determined (11, 12, 17, 26–28). Addition of WBRT together with SRS as well as prior intervention of WBRT followed by SRS as salvage therapy have not been superior to SRS alone (17, 25–29, 67–84). Perez et al. has registered survival boost for SRS alone in comparison to combined approach (30). Univariate analysis showed negative correlation for prior WBRT (HR: 1.58; 1.12–2.22, *p* = 0.0087) while WBRT after SRS was shown to impact survival negatively on multivariate analysis (HR: 1.78; 1.06–2.99, *p* = 0.030) (30). It has been suggested that the better response might have been due to advancement of surgery and chemotherapeutic care of breast cancer patients (26, 85). Breast cancer, not only as the primary site has behaved distinctly, in fact, breast cancer histologic subtypes as primary histology for brain metastases have also been revealed to be distinct entities when it comes to their response to radiation therapy. The prognosis for basal subtype (triple negative), luminal A (ER/PR positive/HER2 negative), HER2 positive/ER/PR negative and luminal B (triple positive) subtype were different in terms of median survival as 7.3, 10, 17.9, and 22.9 months (*p* < 0.01), respectively (31). In a retrospective study of 131 patients who received SRS for breast cancer brain metastases between 2001 and 2013 revealed a median overall survival of 16, 26, 23, and 7 months for ER positive/HER2 negative, ER positive/HER2 positive, ER negative/HER2 positive, and TNBC (triple negative breast cancer), respectively (*p* < 0.001) (32). HER2 positive breast cancer brain metastases responded to SRS better as compared to HER2 negative breast cancer patients (median survival of 31.3 vs. 14.1 months (*p* < 0.01) (33). Patients with TNBC had the shortest time to retreatment with WBRT or SRS or death with hazard ratio of 3.12 (*p* < 0.001) compared to ER positive/HER2 negative (32). Triple negative subtype is associated with worst prognosis after SRS treatment (median survival of TN vs. Non-TN: 6 vs. 16 months) (34). Breast cancer brain metastases, as a whole have good response to SRS alone except for some histology subtypes and the role of WBRT plus SRS could be contested for survival outcome. Control of the primary disease with advancements in surgical and chemotherapeutic interventions might have something to do with the better outcome.

In context of molecular targeted therapy for BM with breast cancer histology, a number of agents have been approved and others are being under investigations. However, agents like transtuzumab (a monoclonal humanized antibody approved for the treatment of HER2-positive breast cancer) has limited intracranial efficacy due to its lack of BBB crossing ability (86). Lapatinib (a dual HER2 and EGFR inhibitor) has also revealed a mere CNS ORR of 6% in a phase II trial of 242 patients that had previously received transtuzumab and radiation therapy (WBRT or SRS) (87). However, in a separate study, patients with concurrent lapatinib had higher rates of complete response (35 vs. 11%, *P* = 0.008) in comparison to SRS alone (88). In patients with HER2-amlified lesions who also had undergone prior radiation therapies (WBRT, SRS and surgery), both HER2 antibodies (17.9 vs. 15.1 m; *p* < 0.04) as well as lapatinib (21.1 vs. 15.4 months; *P* < 5.03) were associated with improved median survival (89). WBRT was associated with better local [LC (SRS+/–WBRT); 6.9 vs. 11.0%, *p* < 0.02] and distant control [DF (WBRT+/–); 17.4 vs. 28.4%, *p* < 0.01] in combination with SRS or other targeted agents (transtuzumab, lapatinib), respectively (90). Concurrent lapatinib and transtuzumab with SRS compared to SRS alone had also significantly improved local control among HER2-amplified lesions [LF (lapatinib); 15.1 vs. 5.7%, *p* < 0.001, and (transtuzumab); 18.4 vs. 10.2%, *p* < 0.003] (90, 91). Yomo et al. also revealed significant 1-year local control in lapatinib group as per lesion analysis but not patient analysis (LC; 86 vs. 69%, *p* < 0.001) (92). Though concurrent use of lapatinib with SRS vs. SRS alone has not yield survival advantage, patients ever using lapatinib were associated with improved median survival (88–91). In retrospective study of 126 BM patients with HER2+ breast cancer undergoing SRS had also received lapatinib (*n* = 47) during the treatment. Use of lapatinib with SRS resulted in significant survival improvement (27.3 vs. 19.5 months, *p* = 0.03) for these patients as opposed to SRS alone (89). A similar result was reciprocated in a separate study with patients ever (*n* = 43) or never (*n* = 41) using lapatinib increased median survival from 23.6 months to 33.3 months (*p* = 0.009) (88). Currently, lapatinib in combination with WBRT or SRS is being investigated (NCT01622868, NCT00470847) which will more clearly establish the role of either radiation therapy in the treatment of HER2 positive breast cancer patients. Lapatinib in combination with capecitabine has also reported a 66% CNS ORR in a study that included 45 RT naïve patients (92). Trastuzumab emtansine (T-DM1) had also reported an intracranial clinical efficacy in 5 out of 10 (50%) patients, suggesting a similar response rate observed with lapatinib plus capecitabine (93). This result is also supported by the retrospective analysis of EMILIA trial that revealed similar PFS (HR = 1.00; *P* = 1.000; median PFS, 5.9 vs. 5.7 months) for patients with baseline brain metastases comparing the two treatments (94). Other agents like afatinib and neratinib had also been tried with mere CNS responses (95, 96). Furthermore, immunotherapy has also entered clinical trial (NCT03449238) in this group of patients in combination with SRS in optimism for synergistic responses.

### Radioresistant Tumors

Melanoma and renal cell carcinoma brain metastases are considered radioresistant, however, their response to SRS have been encouraging (35). Adjuvant WBRT in addition to SRS, in a study of 31 patients with brain metastases from renal cell carcinoma, melanoma, or sarcoma, resulted in 6 month actuarial local control and distant brain failure rate (DBF) of 100 and 17% as compared with 85 and 64% in patients with no WBRT addition (*P* = 0.018 and *P* = 0.0027), respectively. This suggests a role of WBRT in controlling distant failure for these radioresistant histology (36). A Phase II trial of radiosurgery for one to three newly diagnosed brain metastases (*n* = 36) from renal cell carcinoma, melanoma, and sarcoma reported high degree of failures within the brain (~50% of patients by 6 months) with the omission of WBRT (37). Outcome variation among “radioresistant” brain metastases treated with stereotactic radiosurgery was also reported in retrospective study of 189 patients. Survival after SRS was significantly (*P* = 0.0354) worse for patients with melanoma (*n* = 103) and sarcoma (*n* = 9) brain metastases compared to patients with renal cell carcinoma (*n* = 77). The actuarial freedom from progression after 1 year was 64% for renal cell carcinoma patients, 47% for melanoma patients, and 0% for sarcoma patients (*P* < 0.001) (38). Of the radioresistant tumors brain metastases, renal cell carcinoma has resulted in better survival. Overall, a role of WBRT is observed in control of the disease and hence a trial is warranted to identify which primary histology would derive better survival out of the better control.

### Renal Cell Carcinoma

WBRT has achieved survival of only 3.0–4.4 months in RCC brain metastases (39) while a better survival from 9.5 months to as high as 17.8 months have been reported with SRS (36, 40, 41). Primary renal cell carcinoma was identified as predictor of longer survival in a study which also had contained melanoma and sarcoma (36). Addition of WBRT (upfront or as salvage therapy) to SRS was not associated with local control or survival in patients with RCC brain metastases (42). There was no significant difference in distant control either with upfront WBRT [*n* = 2/8 (25%) vs. *n* = 3/9 (33%)] as well (42). A similar scenario of additive WBRT failure in prolonging survival and distant control (46 vs. 50%) was reported in another study (43). Ippen, et al. found significant association (*p* = 0.0097) between prior WBRT with poor overall survival (44). Foakas et al., however, identified that the addition of WBRT to the SRS improved LC (*p* = 0.032) but not OS (*p* = 0.703) (45). Bates et al. also revealed no difference for SRS alone, WBRT alone or WBRT plus SRS in treating RCC patients with BM [8.3 vs. 2.8 m (*p* = 0.82) vs. 8.5 m (*p* = 0.65), respectively] (46). Renal cell carcinoma, though, has derived better survival compared to melanoma, the role of WBRT is not clear. RCC related medical evidence is distinct because of no brain control with WBRT addition.

Though several targeted agents for RCC have been approved so far, their efficacy data in regard to the RCC brain metastases is limited. Sorafenib in combination with radiation therapy used after surgery in a case report of a patient with brain metastasis from RCC revealed a 4-year recurrence free survival (97). Sunitinib has shown safety and efficacy in metastatic RCC in a number of case reports and trials (98–103). Pazopanib and cabozatinib have also demonstrated intracranial activity in this group of patients (104–107). Concurrent multi-kinase inhibitors (mKIs) with SRS have revealed superior median survival (108–110). Significant local control (LF: 93 vs. 60%, *p* = 0.01) and improved median survival (16.6 vs. 7.2 m, *p* = 0.04) was reported in patients with receiving targeted agents in addition to SRS (108). A similar result was achieved in a separate study as well (16.8 vs. 7.3 m, *p* < 0.001) (109). Verma et al. identified TKIs use after BM development was highly significant for deriving survival benefit (23.6 vs. 4.41 m, *p* = 0.0001) (110). Bates et al., however, failed to report such advantage with concurrent TKIs. Nevertheless, radiation therapy in this study comprised of WBRT, SRS or both (46). Sunitinib or other targeted agents mentioned above or immunotherapeutic agents combined with radiotherapy could be evaluated in clinical trials particularly the SRS as suggested by some authors in order to achieve a more potent response as observed in the case of metastatic melanoma (111).

### Melanoma

Use of WBRT alone in treating melanoma brain metastases has merely achieved a median survival of 3.5 months (47). Melanoma brain metastases responds better to SRS, however, median survival reported in the range of 5.3 to 10.6 months is comparatively lower to that of renal cell carcinoma (36, 48–52, 97–101). Up-front WBRT omission was associated with worse overall survival (multivariate HR 2.56, *p* = 0.08), and distant intracranial progression (multivariate HR 2.24, *p* = 0.005) (53). On the other hand, in a study of 185 patients, the addition of WBRT was shown to lack a LC, OS or PFS advantage in these patients (54). Initial WBRT was associated with no survival advantage (*p* = 0.88) but a delay in distant progression was observed; however, not significant (*P* = 0.13; *n* = 6) (55). Role of WBRT cannot be assessed clearly from these studies due to their retrospective nature and the fact that WBRT is usually administered in aggressive disease or in patients with multiple brain metastases (38, 52).

Immunotherapy in the treatment of brain metastases has mainly been assessed in melanoma patients. Recent studies have suggested role of immunotherapy (anti-CTLA-4/anti-PD-1 agents) and targeted therapy (BRAFi/MEKi) in combination with SRS leading to improved survival in comparison to SRS alone (112, 113). SRS followed by immunotherapy or targeted therapy have shown better local control (1-year LC; 100 vs. 83.3%, *p* = 0.023) as well as improvement in survival (MST; 10.95 vs. 2.29 m, *p* < 0.001) (114, 115). A met-analysis of ipilimumab plus SRS revealed significantly better survival over SRS alone in melanoma BMs (112). As well, this benefit from SRS plus Ipilimumab seems to be comparatively superior to ipilimumab combination with WBRT (116). Furthermore, there is theoretical and clinical evidence of this combination leading to brain control other than the therapeutic target area that is also termed as abscopal effect (117, 118). Targeted therapies in melanoma patients aimed at mutation such as BRAF inhibitors (50% of malignant melanomas) and MEK inhibitors have also been studied extensively. Vemurafenib and dabrafenib (BRAF inhibitors) alone or dabrafenib plus trametinib have shown excellent intracranial responses (119–125). Comparative studies have suggested combination of SRS and BRAF inhibitors resulting in better outcome for BRAF-mutant melanoma patients (126–130). Improved local control as well as superior median survival with BRAFi with SRS particularly when used concurrently or initiated after SRS (126–130). Side effects remains a concern as intracranial hemorrhage and radionecrosis has been associated with the SRS in conjunction with BRAF inhibitors (126, 131). These results suggest that this group of patients respond better with SRS in comparison to WBRT even in combination with immunotherapy and targeted molecular agents such as BRAF inhibitors.

It can be suggested that primary histology and subtypes may have a role in defining the outcome in these patients. Hence, pooling them together might have compromised the outcome of the aforementioned primary trials (3–8). In fact, the diverse prognostic factors associated with each histology had led to the creation of diagnosis specific graded prognostic assessment (DS-GPA), which associates different sets of prognostic factors to each histology (13).

## Single Brain Metastases

Previously, Kondziolka et al., suggested omission of WBRT for single brain metastases while denying the existence of micrometastases (not apparent on high-resolution imaging) or diffuse brain disease based on the result of one study (132). However, there is Class I evidence showing a clear role of addition of WBRT reducing the local and distant failure significantly with subsequent improvement in survival. Andrew et al. reported a significant survival for single brain metastatic patients for treatment comparison (WBRT plus SRS vs. WBRT alone) (4). In the overall analysis, SRS boost was not received in some patients (single BM = 15% and 2–3 BM = 24%; 19% overall), which might have also influenced overall survival analysis (4, 13). Post-stratification by DS-GPA of Andrew et al. trial revealed significant survival advantage for favorable recipients (DS-GPA 3.5–4.0) which included single brain metastasis at large (13). Secondary Analysis of the JROSG 99-1 Randomized Clinical Trial revealed significantly improved survival from combined treatment for prognostically better placed recipients (DS-GPA 2.5–4.0) in comparison to SRS alone. Participants were single brain metastases at majority, however, 2–3 and even 4 brain metastases were also present (14). Li et al. reported SRS alone and SRS+WBRT to be better in prolonging life and improving quality of life than WBRT alone for patients with single brain metastasis from lung cancer (16). It is unfortunate that an overall assessment of the single to multiple (2, 3) brain metastases was reported in primary trials but no assessment of treatment comparison for single brain metastasis was carried out except for Andrew et al. The addition of WBRT to SRS seems to essential to treat single brain metastases where a clear advantage in survival is achieved with this approach particularly in comparison to WBRT alone. The significant intracranial control in the majority of the studies and reported better survival for single brain metastasis suggest a diffuse disease state which leads to distant failure when SRS alone is used. SRS alone has been associated with significant local and particularly distant cranial failure and requires high salvage therapy as compared to combined treatment, thereby increasing the number of hospital visits which can increase psychological burden for patients.

### Future Perspective

Treatment paradigms for brain metastases are shifting with the entries of molecular targeted agents and immune checkpoint inhibitors. However, radiation therapy continues to play a role in this group of patients. SRS is taking a more robust role in combination with new agents. However, the local and distant control achieved with combined approach in selected patients might reveal a superior alternative. Or, would the abscopal effect of immunotherapy provide distant brain control associated with additive WBRT? As absence of immunotherapy after radiosurgery (HR: 0.380, *p* = 0.002) increased the odds of developing new brain metastases (50). Moreover, radiation therapy and immunotherapy are still the only options for cancer patients with no harboring mutations. Our study reports a slight clinical benefit for each primary histology with either WBRT or SRS, for example, WBRT plus SRS for NSCLC, SRS for breast cancer and radioresistant tumors like RCC and melanoma. It's encouraging to see that EGFR inhibitors were combined with WBRT for comparison in NSCLC patients with brain metastases and SRS combined with targeted or immunotherapeutic agents is mainly investigated in melanoma and RCC patients. Therefore, to clearly validate the role of each radiotherapeutic approach (WBRT, SRS, or WBRT + SRS) for patients with brain metastases would be essential through a clinical trial with a much more precise selection design based on primary histology along with other influencing factors.

## Author Contributions

All authors listed have made a substantial, direct and intellectual contribution to the work, and approved it for publication.

## Conflict of Interest

The authors declare that the research was conducted in the absence of any commercial or financial relationships that could be construed as a potential conflict of interest.
